# Clinical Practice Guidelines (CPGs) for stroke rehabilitation from Low- and Middle-Income Countries (LMICs): Protocol for systematic review

**DOI:** 10.1371/journal.pone.0293733

**Published:** 2023-11-09

**Authors:** Aditi Hombali, Amreen Mahmood, Dorcas B. C. Gandhi, Sureshkumar Kamalakannan, Nistara S. Chawla, Jennifer D’souza, Gerard Urimubenshi, Ivy A. Sebastian, John M. Solomon, on behalf of the Global Consortium of Stroke Rehabilitation (GCSR)

**Affiliations:** 1 Visible Analytics and Nuffield Department of Primary Care Health Sciences, University of Oxford, Oxford, United Kingdom; 2 Department of Health Professions, Manchester Metropolitan University, Manchester, United Kingdom; 3 Department of Neurology & College of Physiotherapy, Christian Medical College & Hospital, Ludhiana, Punjab, India; 4 Department of Social Work, Education and Community Wellbeing, Northumbria University, Newcastle Upon Tyne, United Kingdom; 5 Department of Physiotherapy, St. John’s Medical College Hospital, Bangalore, Karnataka, India; 6 Department of Physiotherapy, School of Health Sciences, College of Medicine and Health Sciences, University of Rwanda, Kigali, Rwanda; 7 Department of Neurology, St. Stephen’s Hospital, New Delhi, India; 8 Department of Physiotherapy, Manipal College of Health Professions, Manipal Academy of Higher Education, Manipal, Karnataka, India; University of Chichester - Bishop Otter Campus: University of Chichester, UNITED KINGDOM

## Abstract

**Introduction:**

Stroke rehabilitation guidelines promoteclinical decision making, enhance quality of healthcare delivery, minimize healthcare costs, and identify gaps in current knowledge to guide future research. However, there are no published reviews that have exclusively evaluated the quality of existing Clinical Practice Guidelines (CPGs) for stroke rehabilitation from Low- and Middle-Income Countries (LMICs) or provided any insights into the cultural variation, adaptations, or gaps in implementation specific to LMICs.

**Objectives:**

To identify CPGs developed by LMICs for stroke rehabilitation and evaluate their quality using AGREE-II and AGREE-REX tool.

**Methods:**

The review protocol is prepared in accordance with the PRISMA-P guidelines and the review was registered in PROSPERO (CRD42022382486). The search was run in Medline, EMBASE, CINHAL, PEDro for guidelines published between 2000 till July 2022. Additionally, SUMSearch, Google, and other guideline portals and gray literature were searched. The included studies were then subjected to data extraction for the following details: Study ID, title of the CPG, country of origin, characteristics of CPG (Scope-national/regional, level of care, multidisciplinary/uni-disciplinary), and information on stroke rehabilitation relevant recommendations. The quality of the included CPGs will be subsequently evaluated using AGREE-II and AGREE-REX tool.

**Results & conclusion:**

This systematic review aims to explore the gaps in existing CPGs specific to LMICs and will aid in development/adaptation/contextualization of CPGs for implementation in LMICs.

## Introduction

Stroke has consistently been the second leading causes of death and third leading cause of disability in Low-and-Middle-Income-Countries (LMICs) [1, 2]. The evidence base of stroke rehabilitation has grown in the past decade, which is known to improve quality of life, however its application in clinical practice, particularly in relation to rehabilitation is sub-optimal in LMICs [3–5]. It is a common knowledge that Clinical Practice Guidelines (CPGs) promote clinical decision making, enhance quality of healthcare delivery, minimize healthcare costs, and identify gaps in current knowledge to guide future research. Hence, evidence-based CPGs recommend best possible clinical practice [6–9]. Previous reviews have established an association between adherence to CPGs and positive outcomes such as mobility and independence in activities of daily living after stroke [7, 8]. Even though, the evidence suggests implementation of CPGs for better quality of care, it is seen that these are underutilized due to lack of knowledge and skills as well as time and resources constraints among healthcare professionals [10]. Moreover, the lack of specificity, clinical applicability, regional adaptability, knowledge translation and program implementation have caused poorer uptake of CPGs into practice in LMICs [11–13].

In a previous study, it was noted that LMIC CPGs recommend interventions with low evidence, exclude interventions even when its benefits outweigh harms and include recommendations for interventions which have high evidence of hazards [14]. This situation warrants development and/or improvement of CPGs in LMIC as well as implementation and promotion of specific, evidence-based CPGs as a priority to improve quality of stroke rehabilitation in these settings [4, 11, 15].

A rigorous methodology is necessary, but this alone will not facilitate clinical implementation of the CPGs’ recommendations. Previous reviews have discussed recommendations for CPGs in stroke [16–18]. However, till date, no reviews have focused on clinical credibility, trustworthiness and implementability of CPGs from LMICs, giving the opportunity to delve deeper into the nuances of major and subtle variations of LMIC CPGs for stroke rehabilitation. Thus, the need to explore cultural variation, adaptations, or gaps in implementation specific to LMICs which is the focus of the current review. Therefore, the objective of this systematic review is to identify CPGs developed by LMICs for stroke rehabilitation and evaluate their quality using AGREE-II and AGREE-REX tool.

Added value of the study: Firstly, we are not limiting our focus to the AGREE-II & AGREE-REX tools, rather we are looking at the AGREE scores in light of the contextual factors in LMICs that underpin CPG awareness, use and acceptance in addition to the methodological quality and implementability. Secondly, evaluating the AGREE–REX scores depicting practicality/implementability of the CPGs is unique to our review. AGREE-REX is important to assess clinical implementability in LMICs which is different from methodological quality measured by AGREE-II that other reviews have used. Additionally, our methods have been edited from previous reviews to include keyword such as implementation in our search strategy. Our searches were extensive using citation searching, contacting other CPG development groups, grey literature searches via SUMSearch and ministry websites. Lastly, we are inclusive of all types of CPGs whether developed de-novo or contextualized from other CPGs.

## Methods

This review protocol is conducted in accordance with (PRISMA-P) guidelines of systematic review protocols [19]. The checklist for the same is attached as a S1 Checklist. The review protocol is registered in PROSPERO (CRD42022382486).

### Search strategy

To identify keywords, synonyms, free-text words, and controlled vocabulary terms, high-frequency words for ‘stroke’, ‘rehabilitation’ and ‘clinical practice guidelines’ from the pre-selected relevant CPG’s and subject headings from the MESH database were searched. AH and AM independently searched the following electronic databases Medline, EMBASE, CINHAL, PEDro for guidelines published between 2000 till July 2022. SK and DG ran the additional searches in SUMSearch. AH and AM also searched Google, and guideline portals (guidelines international network, National Guideline Clearinghouse, BIGG International database of GRADE guidelines, ECRI Guidelines Trust). Furthermore, a list of groups that are involved in producing CPGs in stroke rehabilitation such as rehabilitation societies or associations existing in LMICs was developed and those groups (WSO and G-score) were contacted to seek information about existing CPGs or ongoing CPGs for stroke rehabilitation. Lastly, websites of stroke association, national and regional health institutes of national importance, and government websites of LMIC were searched.

Moreover, the following additional steps were adopted for searching.

- First Concept: Population (STROKE)
  For stroke, we adapted and modified the search strategy developed by the Cochrane stroke group specialised register [20].
- For the second concept: CPG
  We adapted and modified the search strategy developed by the Canadian Agency for Drugs and Technologies in Health (CADTH’s) database search filter for Guideline that was identified from the InterTASC Information Specialists’ Sub- Group (ISSG) (filter resource website which is found to have high sensitivity in retrieving all CPGs in databases (Medline, EMBASE, CINHAL, PEDro) [3, 4, 15]
- *Searching Google and SUMSearch database*:
  We adopted the GLAD (GuideLine AND disease) search strategy that combines the CPG term with specific disease term with Boolean operator “AND” developed by Haase [11]. For this review purpose we will use “GLAD” “AND” “individual LMIC country name” (Example: “practice guideline” AND “Stroke” AND “Angola”). For this search, all 132 LMICs were identified using the current classification by the World Bank of countries based on the GNI per capita [12].
- Gray literature search documentation:
  Canadian Agency for Drugs and Technologies in Health (CADTH)- created a checklist for documenting gray literature search in line with the international standard. This ensures transparency, reproducibility and conducting search in a comprehensive and structured way [1]. We adopted a similar documenting style for the gray literature search for this review. We also adopted the Cochrane referencing style for gray literature search that includes personal communication, unpublished guidelines, internet sources as shown in Table 1.

**Table 1:** Cochrane referencing style for internet sources, personal communication, and unpublished data.

Unpublished data/guideline		Personal communication: email message	
**Example:** UK/Asia trialists. Individual patient data (as supplied 1 April 1995). Data on file.		**Example:** Smith A. Allocation concealment used in our trial [personal communication]. Email to: C Keystone 27 November 2009.	
**Reference type**	**Unpublished data**	**Reference ID**	**Smith 2009**
		**Reference type**	**Other**
**Authors**	UK/Asia trialists	**Authors**	Smith A
			*Author of the email*
**English title**	Individual patient data (as supplied 1 April 1995)	**English title**	Allocation concealment used in our trial
			[personal communication]
			*Email subject line*
**Journal/book/source**	Email to: C Keystone	**Journal/book/source**	Email to: C Keystone
	*Email recipient*		*Email recipient*
**Date of publication**	27 November 2009	**Date of publication**	27 November 2009
	*Date email sent*		*Date email sent*

### Study selection

**Fig 1:**
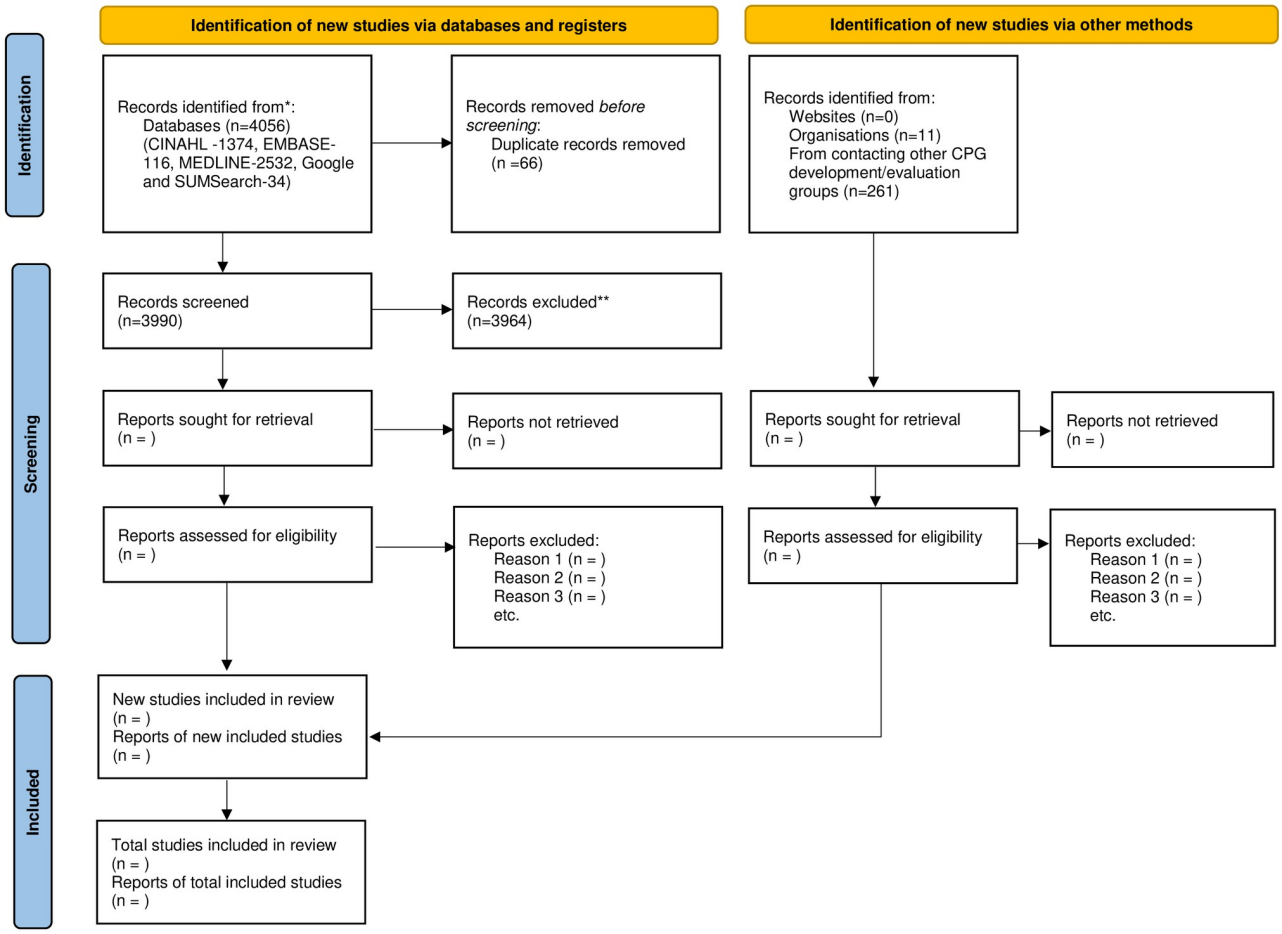
PRISMA 2020 flow diagram for systematic reviews. *Consider, if feasible to do so, reporting the number of records identified from each database or register searched (rather than the total number across all databases/registers). **If automation tools were used, indicate how many records were excluded by a human and how many were excluded by automation tools. From: Page MJ, McKenzie JE, Bossuyt PM, Boutron I, Hoffmann TC, Mulrow CD, et al. The PRISMA 2020 statement: an updated guideline for reporting systematic reviews. BMJ 2021:372:n71. doi: 10.1136/bmi.n71. For more information, visit: http://www.prisma-statement.org/.

The search results retrieved from all the databases were merged using reference managing software Zotero. Duplicate citations were identified and excluded. Searched articles were then imported to Rayyan-intelligent systematic review software. Nine reviewers under supervision of DG and SKK applied the study selection criteria given below to select potentially relevant studies based on title and abstract screening, followed by three independent reviewers (DG, NC, IS) who performed the full text screening. Another independent reviewer resolved any disagreements in study selection. We used Microsoft excel to record decisions of screening along with reasons for exclusion during full text screening. We will report the screening process using a PRISMA flowchart, see Fig 1.

*Inclusion criteria*:

We included the most recent versions of CPG developed for use in LMICs for rehabilitation of individuals with stroke or stroke survivors.

*Exclusion Criteria*:

- CPGs published in languages other than English
- CPGs only available through purchase
- No information on the rehabilitation

### Data extraction

A data extraction form is developed in Microsoft excel. Three reviewers will independently extract data from the included studies on: Study ID, title of the CPG, country of origin, characteristics of CPG (Scope-national/regional, level of care, multidisciplinary/uni-disciplinary), and information on stroke rehabilitation relevant recommendations. The extracted data will then be reviewed for any missing information by two senior authors. Other information on development/contextualization of the CPGs like cost considerations, patient pathway, analysis of health systems, implementation strategies, alternative recommendations, co-designing etc. will be extracted from included papers.

### Data synthesis

The included studies will be narratively synthesized and presented in text and tables to discuss the characteristics, quality, and summary of findings. Since the purpose of this review is to identify and evaluate the quality of CPGs on stroke rehabilitation from LMICs therefore a meta-analysis will not be conducted.

### Quality assessment

Three members of the team assessed and evaluated the quality of included CPGs with Appraisal of Guidelines for Research & Evaluation- II (AGREE-II) [21] and Appraisal of Guidelines for Research & Evaluation- Recommendation Excellence (AGREE-REX) instruments [22]. We will report the scores of each domain on AGREE II and AGREE-REX and narratively describe the relationships within the context of stroke rehabilitation in LMICs.

AGREE II consists of 23 items organized within 6 domains followed by an overall assessment. The six domains include- 1. Scope and purpose, 2. Stakeholder involvement, 3. Rigor of development, 4. Clarity of presentation, 5. Applicability, and 6. Editorial independence. The assessment includes the rating of the overall quality of the guideline and whether the guideline would be recommended for use in practice. Each of the AGREE II items and the two global rating items are rated on a 7-point scale (1–strongly disagree to 7–strongly agree).

AGREE-REX tool consists of 9 items organized within three domains- 1. Clinical credibility, 2. Trustworthiness and 3. Implement-ability. All items are rated using a 7-point scale (1 [lowest quality] to 7 [highest quality]). The overall score will be calculated by adding the scores of nine items and with the formula provided in the AGREE-REX manual.

We have intentionally not mentioned a cut-off for acceptable scores due to the variations that may exist across LMICs with respect to implementation of such CPGs and the determinants that affect implementation. We would rather let the readers decide based on the scores we provide in addition to the qualitative aspects of each CPG. We choose to let readers make the final decision about the usage of CPGs, but we will clearly state the domain scores of each tool and qualitative aspects that may help assist decision making. Those CPGs that score high on the AGREE tools may still be irrelevant to local contexts and thus may not be implementable.

## Discussion & conclusion

This will be the first systematic reviews to identify and evaluate the methodological quality, clinical credibility and implementability of CPGs for stroke rehabilitation from LMICs, therefore, it will aid in recognizing gaps in existing guidelines. The finding of this review will be used for development of a CPG or adaptation/contextualization of an existing CPG for use in LMICs. A collaborative network of experts (GCSR) from various LMICs will come together to develop a CPG specific to LMICs overarching the needs, cultural and regional adaptability, and resource specific recommendations for these countries.

There is a multitude of published guidelines for stroke management, however, very few are from LMICs [18]. A recent WSO guideline collated evidence from existing stroke guidelines across the world and found that in general the guidelines did not consider the resource availability and context for implementation [18]. Authors recommended that stroke services should consider the cost-benefit of any intervention depending on their local resources and circumstances. Therefore, the guidelines and clinical recommendations should factor in the context of different heath care settings [18]. In addition, all relevant stakeholders should be involved during the development/contextualization process to implement effective co-designing [23]. This systematic review would be the first step towards development of a contextualized CPGs for LMICs by identifying the existing guidelines specific to LMICs and appraising their methodological quality for clinical implication.

WSO Global Stroke Service Action Plan classified levels of health service capacity into minimal, essential, and advanced stroke services to ensure that even people who live in minimal resource settings could receive care that could benefit their recovery [24]. Therefore, our next step would be to compare the current evidence-based recommendations for LMIC with the key quality indicators as provided in the WSO roadmap [24].

Despite the evident benefits of stroke guidelines, these are underutilized in clinical practice which compromises patient care and recovery [25–27]. Lack of healthcare professionals’ competence, time constraints, lack of resources and supporting organizational procedures are some of the factors that limit the uptake and utilization of CPGs [10]. Therefore, successful implementation of CPGs requires dissemination of the guidelines, adequate training for healthcare professionals and aligning the organization services to evidence-based recommendations [10]. The findings of this review could be used to promote awareness of the content and quality of the recommendations among healthcare professionals that are specific to their settings and resources.

In future, we aim to contextualize CPGs for LMICs and intend to explore implications of the review by comparing it with accepted clinical standards. This review would also contribute in capacity building by increasing awareness on identification and use of relevant CPGs by rehabilitation professionals.

## Supporting information

S1 Checklist PRISMA-P (Preferred Reporting Items for Systematic review and Meta-Analysis Protocols) 2015 checklist: Recommended items to address in a systematic review protocol*. (DOCX)
